# Extracorporeal Photopheresis: Does It Have a Potential Place Among Cell-based Therapies?

**DOI:** 10.1097/TXD.0000000000001808

**Published:** 2025-09-02

**Authors:** Panagiotis Parsonidis, Thomas Wekerle

**Affiliations:** 1 Division of Transplantation, Department of General Surgery, Medical University of Vienna, Vienna, Austria.

## Abstract

Extracorporeal photopheresis (ECP) is a therapeutic intervention for modulating immune responses using an autologous apoptotic cell-based product, known as a photopheresate. The process of generating photopheresates offers attractive possibilities for manipulating distinct leukocyte subsets to either augment or dampen immune responses, depending on the disease context. This review discusses current uses of ECP as a cell-based therapy and introduces possible strategies to enhance the potency of photopheresates. In Europe, ECP is regulated under the European Union Tissue Directive, but innovative applications in solid organ transplantation, including modifications of the procedure, may force its reclassification as an Advanced Therapy Medicinal Product. Such modifications might include loading dendritic cells with antigens, polarizing dendritic cells toward immunogenic or tolerogenic states, or genetically manipulating leukocyte subsets. In conclusion, although ECP is a long-established, safe, and effective therapy, the more rigorous standards applied to Advanced Therapy Medicinal Product manufacture could help to ensure the quality of photopheresates applied to solid organ transplant recipients.

## Graphical abstract

**Figure:**
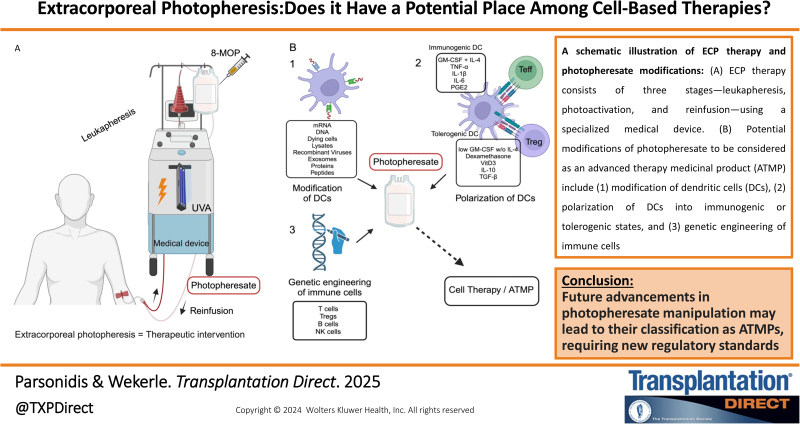

Extracorporeal photopheresis (ECP) is a therapeutic approach that has the ability to modulate immune responses in a range of conditions. Central to this process is the photopheresate, a transfusion product consisting of leukocytes suspended in autologous plasma, which are driven into apoptosis using a photosensitizing agent called 8-methoxypsoralen (methoxsalen) and UVA irradiation. Thus, ECP constitutes a form of autologous cell therapy.^1^ Currently, ECP is approved for clinical use by the US Food and Drug Administration with Premarket Approval (P860003) granted in 1988 as a medical device for the palliative treatment of skin manifestations in patients with cutaneous T-cell lymphoma who have not responded to other forms of treatment, and the approval has been supplemented with updates, the most recent in October 2023, concerning updated devices and components,^2^ and by the European Medicines Agency (EMA) with Orphan Designation (EU/3/06/374) granted in 2006 for the treatment of graft-versus-host disease (GVHD).^3^ However, it is frequently also used off-label in solid organ transplant rejection, especially in heart, lung, kidney, and liver transplantation.^4-6^ Evidence suggests that ECP may be beneficial in both rejection prophylaxis and treatment across various organ types. Although the most robust data exist for cardiothoracic transplantation, immunomodulatory effects of ECP may help reduce reliance on conventional immunosuppressive therapies, potentially minimizing their side effects. Despite variations in treatment protocols and the need for standardized guidelines, ECP has also been explored for reducing posttransplant complications, such as viral infections. However, further high-quality research, including randomized controlled trials, is needed to fully establish its efficacy and optimize its clinical application in solid organ transplantation.^7^ Furthermore, ECP has sporadically found application in treating various dermatologic and autoimmune disorders, including psoriasis, scleroderma, lupus erythematosus, Crohn’s disease, and type 1 diabetes.^8-13^

In September 2023, the classification of cells treated with ECP was informally discussed in the context of scientific recommendations on ATMPs. EMA published the meeting minutes in October 2023.^14^ The Committee for Advanced Therapies (CAT) examined whether the manipulation of autologous blood cells through ECP, using 8-methoxypsoralen (8-MOP) and UVA light, should be regarded as substantial and whether the treated cells could be classified as ATMPs. This discussion revisited earlier deliberations from 2012, which highlighted the absence of a consistent classification across the European Union (EU). A survey conducted by French authorities and the European Commission in April 2021 reaffirmed this inconsistency, with most member states categorizing such procedures under the tissue and cell legislation (Directive 2004/23/EC). CAT members pointed out that 8-MOP treatment primarily eliminates susceptible cells and raised concerns about the challenges in quality controlling these cells before reinfusion. The recommendation of CAT was that in light of the ongoing lack of a unified classification in the EU, national authorities may consider regulating these products under tissue and cell legislation. In contrast, “off-line” photopheresis products are predominantly regulated as Advanced Therapy Medicinal Products (ATMPs) and fall under the approval of pharmaceutical authorities.^15^

Given the clinically approved setting of the ECP procedure, the different regulatory contexts for in-line and off-line photopheresis products highlight the potential for innovation in ECP applications. The manipulation of the photopheresate to enhance therapeutic efficacy appears to be an attractive strategy for future exploration. This review provides an overview of future potential of ECP as a cell-based therapy.

## OVERVIEW OF ECP

**FIGURE 1.**
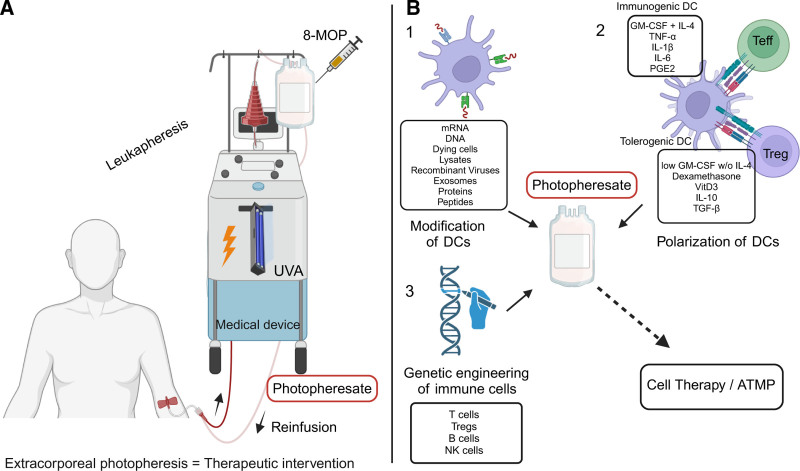
Schematic illustration of ECP therapy and photopheresate modifications. A, ECP therapy and its components. ECP is a therapeutic intervention that involves 3 stages (leukapheresis, photoactivation, and reinfusion). The device using the procedure is a medical device. Photopheresate is the treated buffy coat that has the potential to be characterized as a cell-based medicinal product. B, Potential manipulation of photopheresate to be considered as ATMP: 1, modification of DCs; 2, polarization of DCs into immunogenic or tolerogenic state; and 3, genetic engineering of immune cells. Created with BioRender.com. ATMP, Advanced Therapy Medicinal Product; DC, dendritic cell; ECP, extracorporeal photopheresis; 8-MOP, 8-methoxypsoralen; IL, interleukin; NK, natural killer; T_eff_, effector T; GM-CSF, granulocyte-macrophage colony-stimulation factor; PGE-2, prostaglandin E2; TGF, transforming growth factor; TNF, tumor necrosis factor; Treg, regulatory T cell; UVA light, UV A light; VitD3, vitamin D_3_.

Extracorporeal photopheresis, alternatively referred to as extracorporeal photochemotherapy, is an apheretic therapy initially developed as a treatment for cutaneous T-cell lymphoma.^16^ ECP involves several steps (Figure 1A). A central component of the ECP procedure is the device responsible for cell separation and UVA treatment. The photopheresate is the cell suspension that is collected as buffy coat during the ECP procedure and which is primarily composed of lymphocytes and monocytes and also contains granulocytes and platelets.^17^ The composition and characteristics of ECP products differ notably depending on the system used. The “in-line” system, which is characterized by a shorter runtime, produces products with a higher proportion of mononuclear cells (MNCs) and lower granulocyte content, making it highly automated and efficient. On the other hand, the “off-line” system yields a higher total cell yield, although it is associated with longer procedure times and greater operator intervention.^18^ Although MNC purity and enrichment are significantly higher in the off-line system, no significant correlation has been found between the number of apoptotic cells reinfused and clinical outcomes.^19^ There is currently no definitive recommendation for preferring one system over the other, as the choice is largely determined by institutional preferences, equipment availability, and procedural workflow. For individuals with low MNC counts in peripheral blood, the off-line system may be more suitable, as it allows for higher MNC collection, which has been considered beneficial in some institutional practices.^19^ The off-line approach is generally favored when a high volume of lymphocytes or MNCs per kilogram of body weight is required, or when additional apheresis procedures are routinely used in an institution. In contrast, the in-line system offers advantages when patients have near-normal MNC counts, and time efficiency is a critical consideration.^18^ Despite these differences, there is currently no conclusive evidence establishing a direct link between the cell dose and clinical response in GVHD treatment.^18,19^ This variation in cellular composition may lead to different immunological responses, as highlighted by findings that the identity of apoptotic cells influences macrophage gene expression and function, indicating the potential for tailored macrophage-based therapies.^20^

The ECP procedure begins with obtaining a sample of anticoagulated blood from the patient, drawn via a peripheral or central venous access. Subsequently, the leukocytes are separated from both plasma and nonnucleated cells. With the aid of a centrifugal force, cells are separated and leukocytes are gathered as buffy coat. Then, white blood cells are counted, and the appropriate concentration of the photosensitizing agent, 8-MOP, is calculated on the basis of the cell count. This calculated dose of 8-MOP is then added to the bag containing the buffy coat, and the cells are subsequently exposed to UVA radiation before reinfusion into the patient.^21-23^

Two fundamentally distinct approaches to performing the ECP procedure exist, categorized as “in-line” and “off-line” methods, each characterized by the devices used for leukocyte collection and UVA irradiation. The “in-line” approach involves performing all stages of the procedure (leukapheresis, photoactivation, reinfusion) sequentially within extracorporeal circulation, using a single instrument. Conversely, in the “off-line” method, each phase of ECP occurs sequentially using separate equipment. Leukapheresis is conducted using a continuous flow cell separator with a sterile disposable kit. Collected cells are transferred to a bag where 8-MOP is added. After UVA irradiation, the photopheresate is reintroduced into the patient using a standard transfusion filter. This method is performed in some centers in Europe, whereas in the United States, only the “in-line” method is used.^15,18,24-27^

Mini ECP is a modified version of the classical ECP technique, commonly performed in an off-line setting but also adaptable to an in-line system. It is specifically tailored for children or patients who are unable to undergo traditional ECP due to factors such as low body weight or challenges with central venous catheter access. In Mini ECP, a reduced number of cells are used compared with the standard procedure, making it suitable for patients with limited leukocyte counts or lower total blood volume.^28^

For >35 y, ECP has been used in clinical practice, yet its precise mechanisms remain under active investigation. It has been demonstrated that ECP induces apoptosis through the combination of 8-MOP and UVA light, leading to DNA cross-linking in exposed MNCs.^11,19^ However, the exact mechanisms are still being explored.^29-33^ In ECP, the induction of apoptosis in a high proportion of white blood cells is a key mechanism for achieving therapeutic effects, as apoptotic cells help modulate immune responses through multiple pathways. Studies have shown changes in cytokine profiles with ECP treatment, including reductions in proinflammatory cytokines such as TNF-α and IL-6, and increases in anti-inflammatory cytokines.^34^ ECP treatment reduces monocyte numbers while promoting their differentiation into tolerogenic DCs (TolDCs), which exhibit immunosuppressive properties, such as increased IL-10 production. Additionally, ECP primes monocytes and myeloid DCs for the production and maturation of IL-1β, a key immunostimulatory cytokine, further contributing to the therapeutic effects of ECP through its complex immunomodulatory actions.^35-40^ Efforts are also underway to identify biomarkers for assessing individual therapy responses and to explore new indications for ECP treatments.^41^ Overall, although much progress has been made, further research is needed to fully understand the mechanisms and potential applications of ECP. Given the pivotal role of the photopheresate in eliciting therapeutic responses, further exploration within the context of cell-based therapies is warranted. In addition to these effects, modulation of T cells toward a Th2 phenotype and an increase in regulatory T cells (Treg) have been observed. These Treg are thought to contribute to immune tolerance, particularly in conditions such as GVHD and organ transplantation.^42-46^

## OVERVIEW OF CELL-BASED MEDICINAL PRODUCTS

In key regions such as the United States and the EU—members of the International Council for Harmonization of Technical Requirements for Pharmaceuticals for Human Use—regulations for cell therapy products are structured around specific product categories defined by each jurisdiction. Typically, these products are categorized on the basis of the level of cell manipulation and their intended therapeutic use.^39^ In the United States, therapies involving more-than-minimal manipulation are classified as human cells, tissues, and cellular and tissue-based products, and regulated under Section 351 of the Public Health Service Act (351 HCT/Ps).^40^ Similarly, the EU classifies significantly manipulated therapies as ATMPs.^47,48^ Table 1 provides an overview of the 3 relevant regulations—those for ATMPs, tissue and cell products, and the newly adopted Regulation (EU) 2024/1938—highlighting the critical distinctions in classification and requirements.

**TABLE 1.** Criteria of cell product classification according to Regulation (EC) No 1394/2007 of the European Parliament and of the Council of 13 November 2007 on advanced therapy medicinal products and amending Directive 2001/83/EC, Regulation (EC) No 726/2004 for ATMPs and according to Directive 2004/23/EC for products under tissue and cell legislation, and Regulation (EU) 2024/1938 of the European Parliament and of the Council of 13 June 2024 on standards of quality and safety for substances of human origin intended for human application

Criteria	Regulation (EC) No 1394/2007 (ATMPs)	Directive 2004/23/EC (tissue and cell legislation)	Regulation (EU) 2024/1938 (SoHO)
Definition	Refers to medicinal products intended for human use, encompassing gene therapy, somatic cell therapy, and tissue-engineered products	Focuses on human tissues and cells used for therapeutic purposes	“SoHO” means any substance collected from the human body, whether it contains cells or not, and whether those cells are living or not, including SoHO preparations resulting from the processing of such substance
Gene therapy medicinal product	Involves the transfer of genetic material into cells to modify or restore their function, targeting specific genetic disorders	Not specifically addressed	Can be used to manufacture products regulated by other Union legislation
Somatic cell therapy medicinal product	Use manipulated somatic cells to treat, prevent, or diagnose diseases, focusing on cells that are nonreproductive	General provisions apply to somatic cells, ensuring safety and quality	Can be used to manufacture products regulated by other Union legislation
Tissue-engineered product	Comprises engineered cells or tissues designed to regenerate, repair, or replace damaged human tissues. It may include both human and animal cells, viable or nonviable, and possibly combined with biomaterials or chemical substances	The Directive covers human tissues and cells for therapeutic purposes, including those used in tissue engineering	Can be used to manufacture products regulated by other Union legislation
Nonviable cells/tissues	Products that contain only nonviable cells or tissues, with no significant pharmacological, immunological, or metabolic action, are excluded from the ATMP definition	The Directive mandates quality and safety standards for all human tissues and cells, including nonviable ones used therapeutically	When nonviable SoHO or derivatives are integrated into a medical device, if their action is principal to the device, both the nonviable SoHO and the device are regulated under this Regulation. If their action is ancillary, the Regulation applies only to the SoHO activities before integration, with the final combination subject to Regulation (EU) 2017/745
Manipulation	Cells or tissues that have undergone significant modification to alter their biological characteristics, functions, or structure to achieve specific therapeutic goals	All processing and handling of tissues and cells must ensure their safety and effectiveness	Can be used to manufacture products regulated by other Union legislation
Different functionality in recipient	Cells or tissues intended to perform a different function in the recipient than in the donor are considered engineered	Ensures safety and quality for all tissues and cells, including those that may perform different functions after manipulation	“Effectiveness of SoHO” means the extent to which the human application of SoHO achieves the intended biological or clinical outcome in the SoHO recipient
Combined products	A product that integrates medical devices with cellular or tissue components, where the cellular part plays a primary therapeutic role, is classified as a combined ATMP	The Directive does not directly address combined products.	SoHO can be combined with other regulated products, in particular with medical devices, before human application
Viability of cells/tissues	Viable cells or tissues within ATMPs are primarily responsible for pharmacological, immunological, or metabolic effects	Applies safety and quality standards to all human tissues and cells, whether viable or nonviable, used in therapeutic contexts	Applies safety and quality standards to substances of human origin intended for human application
Autologous vs allogeneic use	ATMPs can involve cells from the same individual (autologous) or another human donor (allogeneic), with specific regulatory considerations for each	The Directive applies to both autologous and allogeneic tissues and cells, ensuring high standards of safety and quality, but excludes certain autologous uses within the same surgical procedure	“Allogeneic use” means the human application of SoHO collected from a person other than the SoHO recipient; “autologous use” means the human application of SoHO collected from the same person
Overlapping definitions	Products that fit within multiple ATMP categories (eg, gene therapy, tissue engineering) are classified according to the most relevant category, such as gene therapy	Not applicable	Not applicable
Characteristics	Novelty, complexity, and technical specificity	Focuses on meeting high safety and quality standards for all tissues and cells used in human applications	Standards of quality and safety for substances of human origin intended for human application
Manufacturing	In compliance with principles of Good Manufacturing Practice	Processing, preservation, storage, and distribution must adhere to high standards to ensure the safety and quality of tissues and cells	This Regulation should always apply to the registration, evaluation, and testing of SoHO donors, as well as to SoHO collection and release. It should also apply to the storage, import, and export of SoHO up to and including their distribution to a manufacturer regulated by other Union legislation

ATMP, Advanced Therapy Medicinal Product; SoHO, substances of human origin.

The recently adopted Regulation (EU) 2024/1938 of the European Parliament and of the Council of 13 June 2024 establishes strict quality and safety standards for substances of human origin (SoHO), including both living and nonliving cells, as well as preparations derived from these substances through processing. It highlights the importance of preventing contamination, ensuring traceability through robust documentation and tracking, and enforcing quality control (QC), particularly for autologous products processed in open systems, where the risk of mix-ups is higher. Although it might be silent for the specific classification of photopheresates, it represents an important update in the regulatory landscape of human-origin therapies. In cases of uncertainty regarding a product’s regulatory status, it is recommended that SoHO competent authorities consult relevant regulatory frameworks and the SoHO Collaboration Board. The SoHO Collaboration Board should ensure harmonized regulatory decisions, supported by the collaboration of all relevant authorities to determine the appropriate classification for each case individually.^49^ Additionally, careful consideration of the Regulation (EU) 2024/1938 is essential when developing processing and QC frameworks for cell therapy products, as it ensures safe handling, quality assessment, and risk mitigation, which are crucial for maintaining both patient safety and product efficacy.

Regulatory approval processes for cell therapy products vary by region, requiring tailored review processes, particularly for clinical trials. The term “cell-based product” is broad, covering a range of therapies, including those classified as ATMPs under European regulations, cell and tissue transplants, and blood cell transfusions.^50,51^ As discussed at the start of the article, the photopheresate is currently categorized under the tissue and cell legislation,^52,53^ but depending on the extent of potential future manipulation steps, it then might be considered an ATMP. This is already the case for products derived from the off-line system, which are classified as ATMPs and require regulatory approval.^15^ Therefore, the focus of this review is on these 2 regulatory categories, which are detailed in the context of relevant criteria, and the classification and regulatory pathways for these therapies, often designed to address significant unmet medical needs. According to Regulation 1394/2007,^54^ a product is classified as an ATMP if it meets specific criteria, such as undergoing substantial manipulation, which alters its biological characteristics, functions, or structural properties, or if it serves a different essential function in the recipient compared with the donor. Navigating the regulatory landscape for these therapies, particularly concerning definitions such as “substantial manipulation” and “same essential function(s),” is crucial for determining appropriate processing and developmental pathways.^55^

Currently approved leukocyte-based cell therapies include dendritic cell and chimeric antigen receptor (CAR) T-cell therapies, both recognized by the Food and Drug Administration and classified as ATMPs by the EMA. For instance, Sipuleucel-T is a dendritic cell vaccine approved for the treatment of prostate cancer. Additionally, 6 CAR T-cell therapies for treating hematological cancers have been approved to date: tisagenlecleucel, axicabtagene ciloleucel, brexucabtagene autoleucel, lisocabtagene maraleucel, idecabtagene vicleucel, and ciltacabtagene autoleucel.^56,57^ Although the approved therapies discussed previously primarily target cancer, current clinical trials are now exploring the use of dendritic cell therapy in the context of transplantation. These trials are investigating the potential of dendritic cell therapy to improve outcomes in transplant procedures. They include a phase I trial (NCT03726307) currently recruiting participants at the University of Pittsburgh, which investigates donor blood monocyte-derived regulatory dendritic cells (DCreg) for renal transplantation. Additionally, 2 phase I/II trials have already been completed, one focused on donor blood monocyte-derived DCreg for liver transplantation at the University of Pittsburgh (NCT03164265), and the other on autologous monocyte-derived DCreg for kidney transplantation as part of the ONE Study (NCT02252055).^58,59^ Moreover, the i-KITCaT study (NCT06243289), which is actively recruiting, investigates the use of TolDCs to mitigate acute kidney injury and improve outcomes in kidney transplantation.^60^

## CURRENT PERSPECTIVES AND FUTURE ENHANCEMENTS OF PHOTOPHERESATE (ECP THERAPY)

The ECP procedure offers perspectives to manipulate autologous leukocytes to enhance therapeutic efficacy. For photopheresates produced through ECP, incorporating manipulation presents unique opportunities and challenges. On the one hand, the in-line system approach ensures a standardized protocol for cell processing, reducing variability related to operator handling and the risk of human error, enhancing the traceability by keeping track of each step in the process, and minimizing the potential for contamination, thereby enhancing the safety of the procedure. On the other hand, the in-line system approach used in ECP poses logistical difficulties, particularly in terms of the time required to perform modifications before reinfusing the cells into the patient. This complexity is further compounded by the challenges identified by the CAT,^14^ particularly regarding the QC of the photopheresate before it is reinfused into the patient, which is crucial for ensuring safety and efficacy.

QC in cell therapies, including ECP, must align with regulations to ensure safety and efficacy before reinfusion. Essential assessments include sterility testing, endotoxin and mycoplasma detection, and evaluation of cell identity, potency, and viability through immunophenotyping, clonogenic assays, and cell counting. Additionally, contamination risks, such as environmental exposure, cross-contamination with pathogens from other donors, and noncellular impurities, must be minimized, particularly in off-line system processes where external handling is involved. Automated QC platforms and QC technologies are increasingly used to enhance reproducibility, reduce manual errors, and optimize cost-effectiveness, further supporting the safety and consistency of cell-based therapies.^61^ Although in-line system approaches in ECP reduce many of these risks, standardized QCs remain crucial to ensure full traceability and maintain the integrity of photopheresates. Ensuring compliance with standardized QCs is essential to maintaining the integrity of photopheresates and safeguarding patient outcomes.

One key aspect of QC is cell viability, which differs significantly between photopheresates and ATMPs. Unlike ATMPs, which often require strict viability criteria to ensure therapeutic efficacy, ECP-treated photopheresates contain large numbers of apoptotic cells, which exert their effects through immunomodulation rather than direct engraftment or expansion. The viability of cells in photopheresates is typically lower than that of ATMPs, as ECP induces apoptosis as part of its (desired) mechanism of action. However, this controlled induction of apoptosis is a therapeutic feature rather than a limitation, as it facilitates the generation of TolDCs and Treg, which are crucial in transplantation and autoimmune disease treatment.

Moreover, although ATMPs often require extensive ex vivo expansion and genetic modifications, photopheresates are processed in a shorter time frame, with minimal manipulation. This difference impacts QC measures, as ATMPs require stringent viability and potency assessments, whereas photopheresates rely more on functional immunomodulatory effects rather than direct cell survival. Further research may help refine the characterization of cell viability thresholds in photopheresates, particularly as their clinical applications expand.

Additionally, further research is needed to assess the feasibility of modifying cells that are on a pathway to apoptosis after 8-MOP and UVA exposure, as this could impact the therapeutic efficacy of the modified photopheresates. Importantly, ECP does not affect all cell types uniformly; circulating lymphocytes are significantly more sensitive to apoptosis induced by 8-MOP plus UVA light compared with monocytes.^62^ Given that monocytes appear more resistant to this form of cell death, they offer a higher potential for manipulation, which could be leveraged to enhance the therapeutic outcome of ECP.^63^ However, the extent of monocyte survival remains controversial. Although some studies indicate that CD14^+^ monocytes selectively persist after ECP, retaining antigen-presenting cell functions and expressing key costimulatory molecules,^39^ others suggest that only a small fraction survive beyond 72 h. Additionally, although monocytes may escape immediate apoptosis, evidence suggests they undergo functional impairment. Compared with UVA-only treatment, ECP-treated monocytes show a reduced ability to respond to stimuli and fail to effectively induce T-cell proliferation.^64^ However, experimental data indicate that monocytes, at therapeutic UVA doses, appear to evade apoptosis,^65^ further supporting the notion that they may persist post-ECP, although their functionality remains in question. Overall, although some findings support monocyte survival after ECP, their long-term viability and functional role remain uncertain, warranting further investigation.

ECP triggers numerous, incompletely defined, biologic processes in the 8-MOP/UVA-treated photopheresate.^66^ One of these processes has been proposed to induce the differentiation of monocytes into DCs, with significant input from activated platelets, in a process that closely mirrors natural physiological mechanisms.^67,68^ DCs serve as key professional antigen-presenting cells, processing and presenting antigens to T cells, thereby orchestrating and regulating immune responses. The capacity of ECP to promote this differentiation and to generate antigen-specific immunity or tolerance underscores its potential as a powerful tool in cellular immunotherapy.^18,69^

Potential interventions that can be envisioned for the future, which would likely lead to the classification of the manipulated photopheresate as ATMPs, include modifications that alter the function and characteristics of immune cells. As illustrated in Figure 1B, these interventions may involve modifying DCs, directing their polarization toward an immunogenic or tolerogenic state, or using genetic engineering approaches to enhance immune cell function. ECP could be viewed as a rapid and safe method for generating therapeutic DCs, with potential applications in cancer treatment, transplant rejection, and autoimmune disorders.^70^ Research has demonstrated that ECP can effectively drive a large percentage of monocytes to differentiate into antigen-presenting DCs within a single day, even in the absence of external cytokines. These DCs are capable of both processing and presenting antigens, showing a high level of uniformity in their maturation as indicated by the consistent expression of key costimulatory molecules. However, the functional outcome of ECP-induced DCs appears to depend on the context. In cancer immunotherapy, studies suggest that ECP promotes the maturation of monocytes into DCs, thereby driving anticancer immunity.^71^ Conversely, in transplantation, where immune tolerance is beneficial, ECP-treated antigen-presenting cells may contribute to regulatory mechanisms that support graft acceptance.^38^ This dual role highlights the adaptability of ECP in different clinical settings and underscores its potential for generating therapeutic DCs tailored to specific immunological needs. The ability of ECP to reliably produce functional DCs could significantly contribute to its clinical effectiveness and underscores its potential as a future source of therapeutic DCs. Enhanced ECP protocols could further align photopheresate-based therapies with ATMPs by incorporating the ex vivo pulsing of DCs with specific antigens tailored to transplantation or autoimmune diseases. This technique, widely explored in immunotherapy, involves pulsing DCs with disease-relevant antigens to modulate immune responses effectively.^72^ Methods for pulsing include the use of mRNA, DNA, dying cells, lysates, recombinant viruses, exosomes, whole proteins, or peptides with specific epitopes.^73^ Thereby photopheresates could be used as innovative tolerogenic or immunomodulatory therapeutic strategies that would meet the complex regulatory standards required for classification as cell-based medicinal products or ATMPs.

Polarizing DCs into either immunogenic or tolerogenic states can tailor immune responses more precisely, particularly in transplantation and autoimmune diseases. TolDCs can suppress harmful immune reactions to disease-specific antigens while preserving overall immune function. The generation of TolDCs from patient-derived monocytes is feasible and shows promise for treating various conditions, including graft rejection, Crohn’s disease, multiple sclerosis, type 1 diabetes, and rheumatoid arthritis.^74-76^ Techniques such as using dexamethasone and low concentrations of granulocyte-macrophage colony-stimulation factor , along with protocols involving IL-10, have been developed to induce a tolerogenic phenotype in DCs. In the context of ECP, directing DCs toward a tolerogenic state could enhance the therapeutic potential of the photopheresate, supporting their classification as cell-based products or ATMPs.^77-79^

Genetic engineering offers a promising avenue for enhancing DC functionality, particularly by improving antigen presentation and modulating immune responses with greater precision. For instance, DCs could be genetically modified to suppress inhibitory molecules such as programmed death-ligand 1 or reduce cytokines such as IL-10, thereby increasing their effectiveness in vivo and encouraging the differentiation of CD8^+^ T cells.^80^ However, unlike T cells, the genetic modification of human DCs remains an emerging field.

CAR T-cell therapy, which uses genetically engineered T cells equipped with CARs, has demonstrated significant potential in immunotherapy.^81^ Beyond oncology, cell-based engineering strategies may also be relevant in the context of transplantation and immune tolerance. Overcoming the limitations posed by the apoptotic pathway in autologous lymphocytes treated with ECP could open the door to applying established genetic engineering techniques to these cells, particularly for modulating immune responses in GVHD and transplantation settings. In the context of managing GVHD after allogeneic hematopoietic stem cell transplantation and CAR T-cell therapy, recent findings suggest that ECP can effectively modulate CAR T-cell function. Specifically, ECP-treated CAR T cells exhibited reduced alloreactivity in mixed lymphocyte reaction assays, likely due to the selective induction of apoptosis, which enriched the population of naive and central memory CAR T cells with lower alloreactive potential. This process was accompanied by a shift in the cytokine environment from immune stimulation to immune tolerance, and importantly, did not compromise the long-term functionality or proliferative capacity of CAR T cells. These results highlight ECP as a promising strategy to mitigate GVHD risk while maintaining the therapeutic efficacy of CAR T cells.^82^ Additionally, the use of CAR in Treg is under investigation for enhancing immune tolerance in transplantation. CAR-Treg could preserve their regulatory phenotype and functionality, effectively migrate to the target tissue, and exhibit superior suppressive capabilities compared with polyclonal Treg.^83^ These advancements could further support the role of ECP-based therapies in immune regulation, reinforcing their relevance in transplantation and autoimmune disease management.

Despite the mentioned limitations, the potential to enhance autologous leukocyte function through genetic engineering within photopheresates, and further development of this technology, could improve the therapeutic efficacy of ECP and support their place among advanced cell-based therapies. If ECP products are classified as ATMPs, they would impose stricter QC protocols, ensuring more consistent product quality and safety, which could expand its reliable use in broader applications, including kidney and liver transplantation, where it is currently less common.

## ETHICAL AND REGULATORY IMPLICATIONS OF CLASSIFYING PHOTOPHERESATES AS ATMPs

Once modified photopheresates need to be classified as ATMPs, several ethical and regulatory challenges are raised. Although conventional photopheresates have a well-documented safety profile under current tissue and cell regulations, their transition to the ATMP category on additional modification would trigger the need for stricter regulatory oversight. This includes compliance with Good Manufacturing Practice (GMP) standards, which are required for ATMPs.^84^ However, the in-line system approach used in the ECP procedure could facilitate adaptation to these stricter regulations, as it inherently supports standardized cell processing and reduces contamination risks, potentially making it more feasible to meet these higher manufacturing standards.

Ethically, the classification of photopheresates as ATMPs presents complex challenges, particularly concerning access and affordability. Traditionally, ATMPs are associated with high production costs due to the complex nature of GMP processes and the extensive clinical trials required for safety and efficacy.^85^ Nevertheless, the closed system manufacturing process used in ECP could potentially offer a more cost-effective solution. By minimizing contamination risks and reducing procedural complexity, ECP might streamline production and lower costs compared with conventional GMP processes. A study analyzing patients with chronic GVHD receiving ECP found that treatment led to a significant reduction in the use of corticosteroids and immunosuppressive agents, alongside a decline in infection rates. Over time, healthcare utilization decreased, with annual healthcare costs dropping substantially from approximately €27 719 in the first year to €1981 by the fifth year. Additionally, productivity loss due to sickness-related workplace absence declined, highlighting potential economic benefits at both the healthcare system and societal levels.^86^ In addition to these long-term cost benefits, the choice of ECP technology can further impact cost-effectiveness. One cost analysis comparing off-line and in-line ECP procedures found that an integrated in-line system significantly reduced procedure duration (from 296 to 120 min) and lowered per-session costs (€1134.57 versus €1063.95). It was estimated that the shift to in-line ECP could save approximately €5932.08 annually while optimizing resource utilization, reducing patient burden, and increasing staff availability.^87^ Beyond these procedural efficiencies, ECP has also been shown to be cost-effective compared with standard-of-care therapies for steroid-refractory chronic GVHD. A cost-utility analysis conducted in Australia demonstrated that ECP resulted in an average cost reduction of $23 999 per patient over a 10-y period while also improving quality-adjusted life-years by 1.10 compared with standard immunosuppressive therapies. These findings were instrumental in securing public reimbursement for ECP in Australia, highlighting its potential to be a sustainable and economically viable option for broader implementation.^88^ These cost advantages could make it easier to provide equitable access to these advanced therapies while managing the increased costs associated with ATMP classification.

However, given the established need for ECP among patients with GVHD and other severe immune-related conditions, changes in regulatory status could have significant implications for patient access. For many patients with GVHD, ECP serves as a critical therapeutic option, especially when other treatments have failed. If the regulatory framework were to change, smaller treatment centers might be unable to offer ECP due to increased regulatory burdens, potentially leaving vulnerable patients without access to this essential therapy. Thus, in addition to ethical considerations, ensuring continued access to ECP for these patients should be a key factor when contemplating the classification of photopheresates as ATMPs.

## CONCLUSIONS

In summary, as advancements in research deepen our understanding of mechanisms and therapeutic potential of ECP, it could potentially advance in the future by exploring new strategies such as targeted manipulation of photopheresates to more effectively modulate immune responses. This might lead to the classification of the modified photopheresate as ATMP. Such a transition would necessitate significant adjustments to meet the regulatory requirements associated with ATMPs.

## ACKNOWLEDGMENTS

Visual abstract and figure were generated with BioRender under the publication license DS27KG29F5.
